# The role of ECMO in COVID‐19: Can it provide rescue therapy in those who are critically ill?

**DOI:** 10.1111/jocs.14635

**Published:** 2020-05-22

**Authors:** Sugeevan Savarimuthu, Jalal BinSaeid, Amer Harky

**Affiliations:** ^1^ Department of Medicine Broomfield Hospital Chelmsford UK; ^2^ Department of Cardiothoracic Surgery Liverpool Heart and Chest Hospital Liverpool UK

**Keywords:** COVID‐19, critical care, ECMO, extracorporeal membrane oxygenation

## Abstract

Arising from the city of Wuhan, Hubei province in China, a novel coronavirus named severe acute respiratory syndrome coronavirus 2 has been rapidly spreading since its first presentation in late 2019. The World Health Organization declared a pandemic on the 11th March 2020, and as of 29th of April 2020 more than 3 million cases have been reported worldwide with over 225 000 confirmed deaths. Where mechanical ventilation may not be enough, extracorporeal membrane oxygenation (ECMO) could play a role as a form of rescue therapy and may provide beneficial results in the hands of skilled clinicians in centers with experience of using ECMO appropriately in selected patients. Our understanding of COVID‐19 is ever‐changing and the need for intensive care beds is rising, which means that ECMO will surely play a key role in the near future.

## 1 INTRODUCTION

Arising from the city of Wuhan, Hubei province in China, a novel coronavirus named severe acute respiratory syndrome coronavirus 2 (SARS‐CoV‐2) has been rapidly spreading since its first presentation in late 2019. The World Health Organization (WHO) declared a pandemic on the 11th March 2020, and as of late April 2020 more than 2.7 million cases have been reported worldwide. We are in unprecedented times fighting a pathogen that is not fully understood. Those who suffer from this illness may experience a clinical course ranging from being asymptomatic to having a minor illness, and in a significant minority a severe form of viral pneumonia, which can result in respiratory failure and death.

SARS‐CoV‐2 is an RNA virus and part of the family of Coronaviruses and the third of its kind to affect humans in recent decades. It is thought to gain entry into type 2 pneumocytes via the ACE2 receptor, and the cytokine storm that is created in response to this virus can result in acute respiratory distress syndrome (ARDS), which can severely impair gas exchange.^1^ Where mechanical ventilation may not be enough to provide sufficient oxygenation, extracorporeal membrane oxygenation (ECMO) may play a role as a form of rescue therapy and may provide beneficial results in the hands of skilled clinicians in centers with experience of ECMO use in appropriately selected patients.^2^

## 2 LITERATURE SEARCH METHOD

A comprehensive literature search is done through the available electronic database to identify articles that reported the use of ECMO and mechanical circulatory support in COVID‐19 patients. The keywords used were “mechanical circulatory,” “extra‐corporeal membrane oxygenation,” “SARS‐CoV‐2,” “SARS‐CoV,” “ECMO,” “VA‐ECMO OR VV‐ECMO” “COVID‐19” “mechanical support.” The search terms were used as keywords and in combination as MeSH terms to maximize the output from literature findings. References of each included article is cross‐checked for any possible relevant study.

The inclusion criteria were articles that discussed the indications, outcomes, and survival benefit from using mechanical circulatory support in the form of VV‐ECMO or VA‐ECMO in patients with COVID‐19.

## 3 WHEN SHOULD WE USE ECMO IN COVID‐19 PATIENTS?

ECMO is a form of cardiopulmonary bypass and can be divided into venovenous (VV‐ECMO) and venoarterial (VA‐ECMO), which can be used in the setting of respiratory failure and cardiogenic shock, respectively. VV‐ECMO can provide respiratory support and can replace the gas exchange function of the lungs and minimize ventilator‐induced lung injury, barotrauma, and oxygen toxicity. VA‐ECMO can provide both respiratory and hemodynamic support and may be of use to COVID‐19 patients who sustain myocardial injury leading to refractory cardiogenic shock.^3^ The WHO has provided guidelines on managing ARDS, which focus on ventilation strategies that have proven to be useful in the setting of ARDS in the past. They advise on using lower tidal volumes (4‐8 mL/kg predicted body weight), lower inspiratory pressure (plateau pressure <30 cmH20), prone ventilation greater than 12 hours, and conservative fluid management, and ECMO usage has been advised in expert centers.^4^

Patient selection is crucial when considering ECMO and those who are inappropriately selected stand a much lower chance of survival. Extracorporeal Life Support Organization (ELSO) has provided selection criteria needed for ECMO referral. If in spite of optimal ventilation strategies, neuromuscular blockade, appropriate PEEP, prone positioning, and the use of pulmonary vasodilators, patients develop the following criteria: PaO2/FiO2 less than 60mm Hg for greater than 6 hours, PaO2/FiO2 less than 50mm Hg less than 3 hours or pH less than 7.20 + PaCO2 greater than 80mm Hg for less than 6 hours, and do not have any contraindications, they may be suitable for ECMO referral.^5^ They have set a list of relative and absolute contraindications such as advanced age, multiorgan failure, advanced lung disease, and severe acute neurological injury to name a few (Table 1). The Murray score (Table 2) stratifies the severity of acute lung injury and values of greater than 2 should be considered for transfer to centers with ECMO facilities. Patients with ARDS and a Murray score of 3 to 4 may be suitable candidates for ECMO.^6^

**Table 1:** Indications for ECMO use in COVID‐19

Indications	(1) Hypoxic respiratory failure despite optimal ventilation strategies (as per ELSO guidelines for ARDS) (2) Severe hypercapnia (pH <7.2 and PaCO2 >80 mm Hg for >6 h) (3) Prolonged ventilation <7 d (4) Cardiogenic shock (refractory to conventional therapy—cardiac index <2 L/min/m^2^, central venous oxygen saturation ScVO2 <65%) (5) Murray score >3 (6) Single organ failure with minimal or no comorbidities
Contraindications	(1) Disseminated malignancy (2) Significant brain injury (3) Irreversible cardiac or pulmonary disease (4) Current intracranial hemorrhage (5) Severe or multiple comorbidities (6) Multiorgan failure (7) Immunocompromised status (8) Advanced age (relative contraindication) (9) Prolonged cardiopulmonary resuscitation >60 min before starting ECMO

*Note*: ARDS, acute respiratory distress syndrome; ECMO, extracorporeal membrane oxygenation; ELSO, extracorporeal life support organization.

**Table 2:** Murray score for acute lung injury

Parameter/score	0	1	2	3	4
PaO2/FiO2 (mm Hg)	>300	225‐299	175‐224	100‐174	<100
CXR	Normal	Alveolar consolidation confined to 1 quadrant	Alveolar consolidation confined to 2 quadrants	Alveolar consolidation confined to 3 quadrants	Alveolar consolidation confined to 4 quadrants
PEEP	<5	6‐8	9‐11	12‐14	>15
Compliance (mL/cmH2O)	>80	60‐79	40‐59	20‐39	<19

*Note*: The final score is calculated by the addition of the component parts. Score: 0, no lung injury; 1‐2.5, mild to moderate lung injury; and >2.5, severe lung injury.

## 4 EFFECT OF ECMO IN PREVIOUS PANDEMICS

Alshahrani et al studied the effect of ECMO during the Middle Eastern respiratory syndrome (MERS) outbreak. Patients suffering from MERS had been known to develop ARDS. VV‐ECMO was offered to those who failed optimal ventilation strategies and were found to have lower in‐hospital mortality, better PaO2/FiO2 ratios, and fewer instances of organ failure.^7^ The use of ECMO has also emerged during the H1N1 pandemic and several studies have reported on their outcomes during that period.^8^ Noah et al^9^ found that mortality rates during the H1N1 pandemic of 2009 were almost twice as much in non‐ECMO patients when compared with ECMO patients; 52.5% and 27.5%, respectively.

When considering ECMO a question of when to intervene arises: in the setting of COVID‐19, would early use of ECMO be beneficial or should patients be placed on mechanical ventilation first and then transitioned to ECMO should they continue to deteriorate? There may be two separate components to hypoxemic respiratory failure: those with normal or high compliance, and those with low compliance and severe hypoxia. ECMO may not be indicated until lung compliance worsens or hypoxemia that is deemed to be severe sets in.^10^

Combes et al placed a group of patients with non‐COVID‐related ARDS immediately on VV‐ECMO and found than after a period of 60 days there was no significant difference between the early application of ECMO and the control group, which followed conventional treatment. Twenty‐eight percent of their patients, however, needed to cross over to the ECMO group for refractory hypoxemia.^11^

## 5 CURRENT EVIDENCE AND OUTCOMES OF ECMO

Our understanding of COVID‐19 is evolving and the role of ECMO is being studied at various specialist centers. These centers tend to save ECMO for those who are critically ill. Yang et al studied 52 critically ill patients and six of these patients were placed on ECMO. Sixty‐seven percent of these patients had ARDS and of the six on ECMO only one had survived beyond the 28‐day period. PaO2/FiO2 ratio was shown to differ between survivors and nonsurvivors being 100 mm Hg and 62.5 mm Hg, respectively, indicating the severity of the disease and prognosis.^12^ Li et al placed eight out of 16 of their COVID‐19 patients on ECMO. Of these four had died (50%) and three were weaned off ECMO but were still on mechanical ventilation (37.5%). Only one patient had been weaned off ECMO before the 28‐day mark.^6^ Zhan et al reported a case of a low‐risk individual who was placed on ECMO therapy within hours of intubation, the clinicians realising early on that mechanical ventilation was insufficient. This patient eventually recovered and was discharged within 40 days of initial presentation.^13^

Henry et al reviewed 234 cases of COVID‐19‐related ARDS in China of which 17 (7.25%) received ECMO. The study showed a 94.1% mortality rate in ECMO patients compared with 70.9% in conventional patients, though the severity of ARDS and the timing of ECMO intervention is not mentioned, bearing in mind that those who are most ill are more likely to be provided with ECMO.^14^ Zeng et al^15^ placed 12 COVID patients on ECMO following hypoxemic respiratory failure despite conventional therapy, four of which have died, and three have been weaned off ECMO and improving. Bemtgen et al presented a COVID‐19 patient with cardiovascular disease and a recent myocardial infarction who was placed on VA‐ECMO and a percutaneous ventricular assist device. The patient developed ARDS, cardiogenic and vasoplegic shock. By day 7 VA was switched to VV‐ECMO and the patient continued to be on ECMO as the ARDS persisted, indicated a role for VA‐ECMO in selected cases, and the persistence and progression of ARDS in COVID‐19 underlining its prognostic value.^16^ Jacobs et al performed a multicenter study involving nine hospitals of the 32 patients that were placed on ECMO. They found 17 patients to be still on ECMO and five survivors successfully decannulated. The average time period from intubation to decannulation was 4 days, and poorer outcomes were seen in patients on VA‐ECMO. Only one out of the 10 who were nonsurvivors suffered cerebral bleeding while on ECMO.^17^

Lung compliance may provide us with a useful parameter to guide us in the use of ECMO. Zochios et al state that in COVID‐related hypoxemic respiratory failure, patients can be divided into those with normal or high lung compliance and those with low lung compliance. ECMO is deemed to be suitable to those who have had prolonged mechanical ventilation (<7 days), worsening lung compliance, and failed conventional strategies.^9^ There have been cases reported of COVID‐19 patients presenting with concomitant pulmonary embolism (PE) and if undiagnosed it may lead to higher mortality rates despite optimal ventilation techniques.^18^

## 6 COMPLICATIONS

Complications can arise from the use of ECMO with hemorrhage and coagulopathy being the most common. Hemorrhage can range from cannula site to intracranial and pulmonary bleeding. These patients are also at risk of thromboembolism paradoxically due to prolonged bed rest, comorbidities, and disease burden. DVT and PE have been identified while patients have been on ECMO. COVID‐19 has been shown to promote thrombosis and in severe cases can result in disseminated intravascular coagulation, which combined with ECMO will place the patient at greater risk of coagulopathy.^19^, ^20^ Mechanical complications though less likely can still occur such as oxygenator and cannula failure. Infection either from cannula sites or nosocomial infections should also be considered and may result in a superimposed infection along with COVID‐19.^21^

## 7 CONCLUSION

ECMO should be reserved for those who are critically ill and in these current times with the emergence of COVID‐19 we are seeing more and more critically ill patients. ECMO is resource‐intensive and requires clinical knowledge, understanding, and experience of ECMO to obtain the best results. There are few intensive care beds and even fewer ECMO machines and it is imperative that patients are selected correctly. ELSO has provided guidance on the criteria needed to refer these patients for ECMO and what cohort of patients should be deemed unsuitable for ECMO. Timing may play an important factor. Should these patients be provided ECMO immediately or soon after presentation, or should maximal ventilation strategies be trialed before ECMO is utilized as a last resort? ELSO currently recommend the latter. Previous viral illnesses in recent times such as MERS and H1N1 have shown to present good results from ECMO. There may be scope for the use of ECMO as rescue therapy and this intervention should, therefore, be strongly considered in patients with severe lung injury secondary to COVID‐19.

## CONFLICT OF INTERESTS

The authors declare that there are no conflict of interests.
